# Predator–prey dynamics of bald eagles and glaucous‐winged gulls at Protection Island, Washington, USA

**DOI:** 10.1002/ece3.5011

**Published:** 2019-03-11

**Authors:** Shandelle M. Henson, Robert A. Desharnais, Eric T. Funasaki, Joseph G. Galusha, James W. Watson, James L. Hayward

**Affiliations:** ^1^ Department of Biology Andrews University Berrien Springs Michigan; ^2^ Department of Mathematics Andrews University Berrien Springs Michigan; ^3^ Department of Biological Sciences California State University Los Angeles California; ^4^ Control and Dynamical Systems California Institute of Technology Pasadena California; ^5^ Department of Computer Science and Mathematics Sul Ross State University Alpine Texas; ^6^ Department of Biological Sciences Walla Walla University College Place Washington; ^7^ Washington Department of Fish and Wildlife Olympia Washington

**Keywords:** Bald eagles, glaucous‐winged gulls, Lotka–Volterra model, predator–prey dynamics, Protection Island, Salish Sea

## Abstract

1. Bald eagle (*Haliaeetus leucocephalus*) populations in North America rebounded in the latter part of the twentieth century, the result of tightened protection and outlawing of pesticides such as DDT. An unintended consequence of recovery may be a negative impact on seabirds. During the 1980s, few bald eagles disturbed a large glaucous‐winged gull (*Larus glaucescens*) colony on Protection Island, Washington, USA, in the Salish Sea. Breeding gull numbers in this colony rose nearly 50% during the 1980s and early 1990s. Beginning in the 1990s, a dramatic increase in bald eagle activity ensued within the colony, after which began a significant decline in gull numbers.
2. To examine whether trends in the gull colony could be explained by eagle activity, we fit a Lotka–Volterra‐type predator–prey model to gull nest count data and Washington State eagle territory data collected in most years between 1980 and 2016. Both species were assumed to grow logistically in the absence of the other.
3. The model fits the data with generalized *R* ^2^ = 0.82, supporting the hypothesis that gull dynamics were due largely to eagle population dynamics.
4. Point estimates of the model parameters indicated approach to stable coexistence. Within the 95% confidence intervals for the parameters, however, 11.0% of bootstrapped parameter vectors predicted gull colony extinction.
5. Our results suggest that the effects of bald eagle activity on the dynamics of a large gull colony were explained by a predator–prey relationship that included the possibility of coexistence but also the possibility of gull colony extinction. This study serves as a cautionary exploration of the future, not only for gulls on Protection Island, but for other seabirds in the Salish Sea. Managers should monitor numbers of nests in seabird colonies as well as eagle activity within colonies to document trends that may lead to colony extinction.

## 1 INTRODUCTION

After years of decline, bald eagle (*Haliaeetus leucocephalus*) populations throughout North America rebounded in the latter part of the twentieth century following tightened protection, reduction in the use of lead shot by hunters, and the outlawing of pesticides such as DDT (Hipfner et al., 2012; Watson, Stinson, McAllister, & Owens, 2002). This recovery has provided one of the great success stories of the conservation movement (Millar & Lynch, 2006). Nowhere has recovery been more pronounced than in the Pacific Northwest of North America where inland waterways such as the Salish Sea, Columbia River, and scores of smaller lakes and streams provide ideal perching, hunting, and nesting opportunities for these raptors (Elliott, Elliott, Wilson, Jones, & Stenerson, 2011; Stinson, Watson, & McAllister, 2001; Watson, 2002; Watson et al., 2002).

An unintended consequence of bald eagle recovery has been the negative impact on seabirds, which are already stressed by overfishing, gill netting, and habitat destruction (Atkins & Heneman, 1987; Blight, Drever, & Arcese, 2015). Although populations of some seabirds may be declining to historic levels (Elliott et al., 2011), local populations of seabirds such as common murres (*Uria aalge*) may be threatened (Parrish, Marvier, & Paine, 2001). Numbers of salmon and other fish traditionally eaten by wintering bald eagles have plummeted in recent years, affecting eagle survival and possibly resulting in their shift to other food sources (Elliott et al., 2011). Seabirds always have formed part of the diet of eagles (Stalmaster, 1987), but increasing numbers of eagles and concurrent prey fish shortages have resulted in increased eagle foraging on waterfowl, a cause for concern among ornithologists (Elliott et al., 2011; Hipfner et al., 2012; Moul & Gebauer, 2002; Parrish et al., 2001; Sullivan, Hazlitt, & Lemon, 2002; Vennesland & Butler, 2004; White, Heath, & Gisborne, 2006). Potential impacts of bald eagle populations on marine food‐web structure appear to be due to resident eagles, rather than overwintering eagles, and the rates at which they consume seabirds as prey (Harvey, Good, & Pearson, 2012).

Bald eagles can impact seabirds both directly and indirectly (Hipfner et al., 2012; Parrish et al., 2001). The most obvious direct effect is the killing and eating of adults, juveniles, and eggs (DeGange & Nelson, 1982; Hayward, Galusha, & Henson, 2010; Hayward, Gillett, Amlaner, & Stout, 1977). A second direct effect is the extra expenditure of energy needed for nesting or feeding in the presence of eagles (Henson et al., 2012; Parrish et al., 2001). An indirect effect results when disturbances displace breeding adults from their nests and expose unprotected eggs and young to other predators (Hayward et al., 2010). A second type of indirect effect involves changes in distribution patterns in response to the presence of eagles. For example, diving waterbirds in the Strait of Georgia moved away from inshore waters, and dabbling ducks formed larger aggregations inshore and were more vigilant, in response to increased eagle presence (Middleton, Butler, & Davidson, 2018).

From 1900 to the early 1980 s, breeding populations of glaucous‐winged gulls (*Larus glaucescens*) markedly increased in the Georgia Basin of the Salish Sea, British Columbia. By 2010, populations had declined to about 50% of peak levels (Blight et al., 2015; Sullivan et al., 2002). A study that incorporated more southern areas of the Salish Sea also reported overall declines from 1975 to 2007 (Bower, 2009). Protection Island, Washington, located in the southeastern Strait of Juan de Fuca and centrally positioned in the Salish Sea, has functioned as a breeding center for marine birds since at least the 1940s (Power, 1976). A large glaucous‐winged gull colony had become established by the early 1960s (Richardson, 1961). Today, Rhinoceros auklets (*Cerorhinca monocerata*), glaucous‐winged gulls, pigeon guillemots (*Cepphus columba*), and harbor seals (*Phoca vitulina*) breed there in large numbers. Adult auklets, gulls, and guillemots, as well as the eggs and chicks of gulls and the afterbirths and pups of seals, all serve as food for nesting and visiting eagles (Cowles, Galusha, & Hayward, 2012; Hayward, 2009; Hayward et al., 2010).

During the 1980s, few eagle disturbances of the gull colony on Protection Island's Violet Point were noted, and from 1980 to 1993 gull nest numbers increased by 37% (3,796–5,189; Table 1). Beginning in the 1990s, however, a dramatic rise in bald eagle activity over and within the colony was observed (Galusha & Hayward, 2002; Hayward et al., 2010), with a significant decline in numbers of breeding gulls at the site (Cowles et al., 2012). Bald eagles constitute the only significant source of interspecific predation on the gulls in this colony (Hayward et al., 2014). The decline of the Violet Point gull population began about 1990 (Table 1), slightly later than declines for the Salish Sea generally (Blight et al., 2015), but otherwise Violet Point trends paralleled those reported for the region. Although systematic counts of gulls nesting on the upper plateau of Protection Island have not been made, nests are now absent from several areas that once contained nesting gulls and nesting has not expanded into other areas of the island (J. L. Hayward, unpublished observations).

The dynamics of the Violet Point gull colony beg two questions. First, is the observed decline caused, at least in part, by eagle activity? Second, are this and other seabird populations merely declining to historic levels, or are their fates less certain? In this study, we use mathematical modeling techniques to investigate whether the dynamic trends in numbers of gull nests on the Violet Point colony can be explained by the dynamics in numbers of occupied eagle territories in Washington State, a proxy for numbers of eagles on Protection Island, and whether there is an approach to stable coexistence for gulls and eagles.

**Table 1:** Observed data

Year	Gull Nests, Protection Island	Occupied Eagle Territories, WA	Observed Eagles, Protection Island
1980	3,796	105	
1981		126	
1982	4,068	138	
1983		168	
1984	4,726	206	
1985		231	
1986		250	
1987	4,958	268	
1988		309	
1989	5,045	369	
1990		403	
1991	4,551	445	
1992		468	
1993	5,189	493	9
1994		547	8
1995		558	19
1996		594	16
1997	4,278	582	16
1998		666	24
1999			26
2000			17
2001		673	12
2002	2,472		23
2003			
2004	2,925		
2005		840	38
2006	2,281		
2007			
2008	2,830		
2009	3,018		
2010	2,495		
2011	2,364		
2012	2,093		
2013	1,850		
2014	1,589		
2015	1,832		
2016	2,512		

## 2 MATHEMATICAL MODEL

### 2.1 Classic Lotka–Volterra predator–prey model

**Figure 1:**
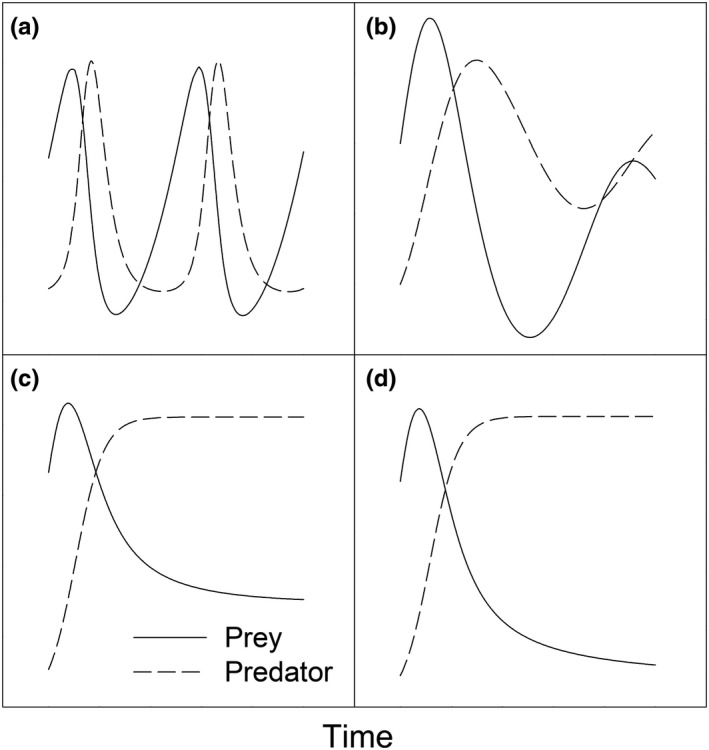
Predator–prey dynamics. (a) Classic predator–prey cycles. (b) Coexistence approached through damped oscillations. (c) Coexistence approached in a nonoscillatory fashion. (d) Extinction of prey

In their classic paper on Canada lynx (*Lynx canadensis*) and snowshoe hare (*Lepus americanus*) populations, Elton and Nicholson (1942) used 100‐year records from the Hudson Bay Company on the numbers of pelts purchased from trappers. The classic predator–prey cycles of theoretical ecology (Figure 1a), often illustrated with lynx‐hare data, are produced by the Lotka–Volterra predator–prey ordinary differential equation model (Henson, 2012; Lotka, 1925; Volterra, 1926)

(1) $$\begin{array}{c} G^{\prime }=aG-\alpha GE\\ E^{\prime }=-bE+\beta GE. \end{array}$$

Here the “prime” denotes the derivative with respect to time, *G* and *E* refer to numbers or densities of prey (gulls, in this context) and predators (eagles), *a* > 0 is the per capita growth rate of the prey population in the absence of the predators, *b* > 0 is the per capita decline rate of the predator population in the absence of prey, *α* > 0 is the predation rate (the probability per unit time that a given prey individual will be taken by a given predator), and *β* > 0 is the conversion rate of prey into predators.

The classic predator–prey model (1) has two major deficiencies. First, the prey population grows exponentially, without bound, in the absence of predators; and second, the predator population declines exponentially to extinction in the absence of the prey. Neither of these scenarios is feasible in most ecological communities because population growth is always eventually bounded by self‐limitation, and predators usually can switch prey and hence do not decline to extinction with the removal of a single prey species. This is true for bald eagles, which are considered opportunistic foragers (Buehler, 2000).

### 2.2 Gull‐eagle predator–prey model

To examine the relationship between the Violet Point gull colony and eagle activity in terms of a predator–prey interaction, we modified the Lotka–Volterra predator–prey model (1) to include a multiple prey base for eagles and self‐limitation terms for both gulls and eagles. In particular, we used the Lotka–Voterra‐type ordinary differential equation model (Ricklefs, 1990)

(2) $$\begin{array}{c} G^{\prime }=rG-\frac{r}{K}G^{2}-\alpha GE\\ E^{\prime }=sE-\frac{s}{C}E^{2}+\beta GE, \end{array}$$

where *G* and *E* are the numbers of gull and eagle pairs, respectively, rather than individual animals as in the original Lotka–Volterra model. Here *r* > 0 and *s* > 0 are the inherent per capita growth rates for gulls and eagles, respectively, at small population sizes, and *r/K* > 0 and *s/C* > 0 are their rates of self‐limitation. The parameter *α *> 0 denotes the predation rate of eagles on gulls, and *β* is the conversion rate of gulls into eagle births. In the absence of the other species (when *α *= *β* = 0), each species grows logistically with carrying capacities *K* > 0 for gulls and *C* > 0 for eagles.

Model (2) does not predict sustained predator–prey cycles; rather, it predicts only equilibrium dynamics. Derivations of the equilibria and stability for model (2) are shown in Appendix A; here we simply summarize the possibilities.

Model (2) has four equilibrium states: the extinction equilibrium (0, 0) in which both species are absent; an equilibrium (*K*, 0) in which eagles are absent and gulls are at their carrying capacity *K*; an equilibrium (0, *C*) in which gulls are absent and eagles are at their carrying capacity *C*; and a coexistence equilibrium ($\overset{¯}{G},\overset{¯}{E}$) with gull and eagle numbers given by

(3) $$\overset{¯}{G}=\frac{sK\left(r-\alpha C\right)}{\alpha \beta KC+rs}\;\text{and}\;\overset{¯}{E}=\frac{rC\left(s+\beta K\right)}{\alpha \beta KC+rs}.$$

There are two main dynamic alternatives:

- If *r* > *αC*, then both $\overset{¯}{G}$ and $\overset{¯}{E}$ are positive in Equation (3), and so the coexistence state (3) is biologically feasible. In this case, the equilibria (0, 0), (*K*, 0), and (0, *C*) are unstable and the coexistence equilibrium ($\overset{¯}{G}$,$\overset{¯}{E}$) is stable. This equilibrium is either a stable spiral or a stable node. That is, gulls and eagles either approach the coexistence equilibrium through damped predator–prey oscillations (1b), or else they approach equilibrium in a nonoscillatory fashion. In the latter case, early transient dynamics may resemble predator–prey oscillations, but the oscillations do not persist (1c).
- If *r* < *αC*, the coexistence equilibrium (3) is not biologically feasible (because the equilibrium number of gulls $\overset{¯}{G}$ is negative). The equilibria (0, 0) and (*K*, 0) are still unstable, but the equilibrium (0, *C*) in which gulls are absent and eagles are at carrying capacity *C* is now stable. That is, gulls approach extinction, whereas eagles approach their carrying capacity *C*. Early transient dynamics may resemble predator–prey oscillations before gulls eventually go extinct (1d).

The biological interpretation of these alternatives is the following. The number *r* is the inherent net reproductive rate of gulls, and the number *αC* is the rate at which gulls are taken by *C* eagle pairs. If the inherent net reproductive rate of gulls is larger than the rate at which gulls can be taken by *C* eagle pairs, then gulls and eagles both survive and approach a positive coexistence equilibrium. If, however, the inherent net reproductive rate of gulls is smaller than the rate at which gulls can be taken by *C* eagle pairs, then gulls go extinct and eagles approach their carrying capacity *C*.

## 3 MATERIALS AND METHODS

### 3.1 Gull nest count data for Violet Point, Protection Island

**Figure 2:**
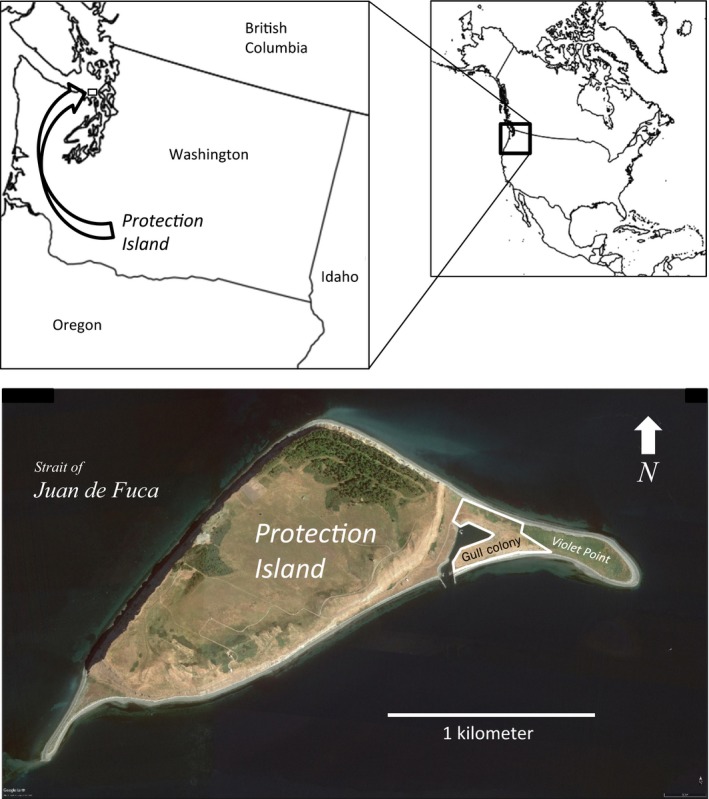
Location of Protection Island and the Violet Point glaucous‐winged gull colony (enclosed by white polygon) at the southeast end of the Strait of Juan de Fuca, Washington. Upper line maps created with Simple Mappr and aerial photograph of island created with Google Earth Pro

We used glaucous‐winged gull nest count data collected between 1980 and 2016 at a large breeding colony on Violet Point, Protection Island National Wildlife Refuge, Washington (48°07′40″N, 122°55′3″W), which lies at the eastern end of the Strait of Juan de Fuca in the Salish Sea (Figure 2). The Violet Point colony is populated by glaucous‐winged gulls and glaucous‐winged gull × western gull (*L. occidentalis*) hybrids (Bell, 1996, 1997). Most of these hybrids resemble glaucous‐winged gulls more than western gulls (Megna, Moncrieff, Hayward, & Henson, 2014; Moncrieff, Megna, Hayward, & Henson, 2013); hence, we refer to these birds collectively as glaucous‐winged gulls.

Gull counts from 1980 through 2014 were carried out in squad fashion as described in Galusha, Vorvick, Opp, and Vorvick (1987). A line of human counters, spaced at distances appropriate for nest density and visibility, moved forward over the colony, with each counter tallying all nests between herself or himself and the next counter. After a distance of 20–30 m, counter tallies were summed and recorded, and the process was repeated until the entire colony was covered. The 2015 and 2016 counts were made by mapping the position of each nest with ArcGIS Desktop 10 using data collected by a Trimble 6,000 Series GPS. Table 1 contains the following corrections and additions from previously published values: (a) Counts published by Galusha et al. (1987) inadvertently omitted some sections of the colony that had been counted, and we added counts for these sections; (b) counts for 2008–2010 reported by Cowles et al. (2012) did not include counts of nests bordering the west shore of the marina (Figure 2), absent before 2008, which we now added (J. G. Galusha, unpublished data); (c) counts for 2011–2015 are newly reported; and (d) counts for 2013, 2015, and 2016 include estimates of uncounted nests bordering the west shore of the marina derived from linear interpolation based on the 2008–2012 and 2014 counts for that area.

### 3.2 Occupied eagle territory data for Washington State

We obtained data for the number of breeding territories occupied by bald eagles each year from 1980 to 1998 in Washington State from Watson et al. (2002). We obtained eagle occupancy data for 2001 and 2005 from the Wildlife Resource Data System of the Washington Department of Fish and Wildlife, Olympia, Washington (Table 1).

### 3.3 Counts of nesting and non‐nesting eagles on Protection Island

We obtained annual maximum numbers of subadult and adult bald eagles observed simultaneously on Protection Island from 1993 through 2002 (Table 1) from Hayward et al. (2010). Eagle counts for 2005 were from unpublished observations (J. L. Hayward, unpublished data) made in the same way as those previously published in Hayward et al. (2010).

### 3.4 Washington eagle territories as a proxy for eagles on Protection Island: correlation analysis and Poisson regression

Numbers of occupied eagle territories in Washington State were larger and relatively less noisy than numbers of eagles observed on Protection Island. Hence, for model fitting we wished to use a scaled version of the statewide eagle data as a proxy for the Protection Island eagle data. To determine whether we could use the numbers of occupied eagle territories in Washington State as a proxy for eagle activity on Protection Island, we performed a correlation analysis on the number of eagles observed on Protection Island and the number of occupied eagle territories in Washington State for the eight years these data overlapped (1993–1998, 2001, 2005; Table 1). We considered a strong positive correlation (*ρ* ≥ 0.60), if significant at the 0.05 level, a justification for using the proxy in further analyses. Because our dependent variable involved count data, we used Poisson regression to obtain the proxy equation that predicts the number of eagles observed on Protection Island as a function of the statewide number of eagle territories. We used the glmfit function in matlab ^®^ (MathWorks™, R2012a) with dispersion to obtain the Poisson regression equation relating the two quantities. The dispersion parameter is estimated in order to increase the *P*‐values appropriately if the data are overdispersed.

### 3.5 Model parameterization

Ecologists must consider several factors when fitting theoretical models to time series data. Populations are subject to both demographic and environmental stochasticity resulting in “process noise” (Dennis, Ponciano, Lele, Taper, & Staples, 2006). Inaccuracies in the estimates of population densities also result in measurement error (Carpenter, Cottingham, & Stow, 1994). Although methods have been developed to deal with these factors (e.g., Valpine & Hastings, 2002), the data demands for these methods are sometimes prohibitive. In particular, the data used here raise challenges for model parameter estimation. There are gaps in the gull and eagle time series, and in some years, data are not available for both species. The sample sizes differ for gulls and eagles, and the numbers for the two species are different in magnitude. These difficulties preclude the application of techniques based on autoregressive time series that underlie many of the methods and software commonly in use (Bolker et al., 2013).

To estimate the parameters in model (2), we first scaled the state variables *G* and *E*, as well as the data, by dividing by the observed standard deviations *σ*_g_ and *σ*_e_, respectively (that is, $\hat{G}=G/\sigma_{g}$ and $\hat{E}=E/\sigma_{e}$), to scale the data to comparable magnitudes. It follows that the derivatives are ${\hat{G}}^{\prime }=G^{\prime }/\sigma_{g}$ and ${\hat{E}}^{\prime }=E^{\prime }/\sigma_{e}$, and so one can rewrite model (2) in terms of the scaled variables:

(4) $$\begin{array}{c} {\hat{G}}^{\prime }=r\hat{G}-\frac{r\sigma_{g}}{K}{\hat{G}}^{2}-\alpha \sigma_{e}\hat{G}\hat{E}\\ {\hat{E}}^{\prime }=s\hat{E}-\frac{s\sigma_{e}}{C}{\hat{E}}^{2}+\beta \sigma_{g}\hat{G}\hat{E} \end{array}$$

To fit model (4) to the scaled data, we used the ode45 differential equation solver in matlab ^®^ to produce predicted model trajectories from 1980 to 2016. We treated the (scaled) initial conditions ${\hat{g}}_{0}$ and ${\hat{e}}_{0}$ as parameters to be estimated. Given a vector of parameter estimates $\theta =\left({\hat{g}}_{0},{\hat{e}}_{0},r,K,\alpha ,s,C,\beta \right)$, we computed residuals on the log scale to account for environmental stochasticity, which is approximately additive on the log scale (Cushing, Costantino, Dennis, Desharnais, & Henson, 2003; Dennis, Munholland, & Scott, 1991):

(5) $$\begin{array}{c} \gamma_{t}=\ln\left({\hat{G}}_{t}\right)-\ln\left({\hat{g}}_{t}\left(\theta \right)\right)\\ \varepsilon_{t}=\ln\left({\hat{E}}_{t}\right)-\ln\left({\hat{e}}_{t}\left(\theta \right)\right) \end{array},$$

where ${\hat{g}}_{t}\left(\theta \right)$ and ${\hat{e}}_{t}\left(\theta \right)$ are predicted values obtained by numerically integrating model (4) from year 1980 to 2016, using parameters *θ*, the parameters ${\hat{g}}_{0}$ and ${\hat{e}}_{0}$ as initial conditions, and the observed standard deviations *σ*_g_ and *σ*_e_ for gulls and eagles, respectively. We obtained best fit parameters $\tilde{\theta }$ by minimizing the sum of the root mean squares (*RMS*) of the residuals

(6) $$RMS\left(\theta \right)=\sqrt{\frac{\sum_{t=1}^{n_{g}}{\gamma_{t}}^{2}}{n_{g}}}+\sqrt{\frac{\sum_{t=1}^{n_{e}}{\varepsilon_{t}}^{2}}{n_{e}}},$$

as a function of *θ*, where *n*_g_ and *n*_e_ are the number of residuals for gulls and eagles, respectively, using the fminsearch downhill search algorithm in matlab ^®^.

The fitting method described above is based on a number of considerations. First, the two time series are not paired; in many years, estimates are available for only one of the two species. Therefore, we cannot view the data for every year as a traditional bivariate observation which would allow a traditional sum of squared errors, and we also cannot use one‐step predictions in computing residuals. Second, each species has different numbers of observations, so the sum of squared residuals must be scaled by the number of observations. Otherwise, the parameter estimates would bias the species with the most observations. Third, we cannot estimate parameters using separate RMS values because the equations are coupled, so the fit of one species affects the fit of the other. Fourth, the overall magnitudes of the two species differ; hence, we scaled the data by the standard deviations so that the two terms in the RMS equation would be commensurate and the parameter estimates would not be biased in favor of fitting the species with overall higher numbers.

We performed diagnostic analyses of the gull and eagle residuals to check for independence and normality. We plotted the residuals as a function of time and examined normal quantile–quantile plots for departures from normality. We computed first‐ and second‐order autocorrelations of the gull and eagle residuals that were separated by one and two years, respectively, and tested these correlations for significance. We also computed the Shapiro–Wilk test statistic for normality (Shapiro & Wilk, 1965).

### 3.6 Goodness‐of‐Fit

We used a generalized *R* ^2^ to check the goodness of fit of the scaled model (4) to the scaled data:

(7) $$R^{2}=1-\frac{\text{RM}S_{M}}{\text{RM}S_{T}}.$$

Here *RMS_M_* is the fitted root mean square using model (4) as the predictor and using Equation (5) to compute the residuals, whereas *RMS*_T_ is the sum of the root mean squares using the central tendency (mean) of the data as the predictor and using the following equation to compute the residuals in Equation (6) for *RMS*_T_:

(8) $$\begin{array}{c} \gamma_{t}=\ln\left({\hat{G}}_{t}\right)-\text{mean}\left[\ln\left({\hat{G}}_{t}\right)\right]\\ \varepsilon_{t}=\ln\left({\hat{E}}_{t}\right)-\text{mean}\left[\ln\left({\hat{E}}_{t}\right)\right] \end{array}.$$

We also computed the adjusted goodness‐of‐fit *R*_A_^2^ by

(9) $${R_{A}}^{2}=1-\frac{n_{g}+n_{e}-2}{n_{g}+n_{e}-p-2}\left(1-R^{2}\right),$$

where *p* = 8 is the number of estimated model parameters. In general, *R*_A_^2^ is smaller than *R* ^2^ because it takes into account the number of estimated parameters and penalizes the goodness of fit as *p* increases. A version of Equation (9) is used in multiple regression models, but in that case one uses −1 instead of −2 (Zar, 2009). Here we have two means (for gulls and eagles) instead of one mean, so we reduce the degrees of freedom by one more unit. Equations (7) and (9) represent a “generalized” coefficient of determination (Anderson‐Sprecher, 1994), not the traditional value used in linear regression.

### 3.7 Confidence intervals for parameters

Once a deterministic model has been fitted to population time series data, bootstrapping methods can be used to obtain confidence intervals for the estimated parameters (Dennis, Desharnais, Cushing, Henson, & Costantino, 2001; Falck, Bjornstad, & Stenseth, 1995). We randomly sampled, with replacement, from the model residuals $\gamma_{1},\gamma_{2},\cdots ,\gamma_{n_{g}}$ and $\varepsilon_{1},\varepsilon_{2},\cdots ,\varepsilon_{n_{e}}$ to create sets of surrogate residuals $\gamma_{1}^{*},\gamma_{2}^{*},\cdots ,\gamma_{n_{g}}^{*}$ and $\varepsilon_{1}^{*},\varepsilon_{2}^{*},\cdots ,\varepsilon_{n_{e}}^{*}$. The time order of the residuals was ignored when sampling (Dennis et al., 2001; Falck et al., 1995).

The surrogate residuals were used to create surrogate data:

(10) $$\begin{array}{c} {\hat{G}}_{t}^{*}={\hat{g}}_{t}\left(\tilde{\theta }\right)\exp\left(\gamma_{t}^{*}\right)\\ {\hat{E}}_{t}^{*}={\hat{e}}_{t}\left(\tilde{\theta }\right)\exp\left(\varepsilon_{t}^{*}\right) \end{array}.$$

For each surrogate data set, we estimated point parameters, using the method explained in section 3.6. This process was repeated *n_S_* = 2,000 times using an independent random sampling of the original residuals for each iteration. If the fminsearch algorithm did not converge to a solution within 1,000 functional evaluations, these steps were repeated for a new set of surrogate data. This occurred at a rate of 21.6%. The lack of convergence in some of the bootstrap realizations was due to the fact that the time series are relatively short and, consequently, the overall number of residuals is small, frequently leading to sets of resampled residuals that negatively impact the rate of convergence for the minimization algorithm. We independently repeated the analyses several times with only trivial variations in the results to verify that 2,000 repetitions were adequate and that the nonconvergent bootstrap realizations were not a problem.

This procedure yielded a set of bootstrapped parameter estimates $\theta_{1}^{*},\theta_{2}^{*},\cdots ,\theta_{n_{s}}^{*}$ that should reflect the variation one would see in the best fit parameters assuming the model (4) is valid, and the observed residuals from the model are random effects with no autocorrelation or cross‐correlation.

The 95% confidence intervals for the point parameter estimates were obtained by ranking the parameter estimates for the surrogate data sets and computing the 2.5th and 97.5th percentiles (Dennis et al., 2001).

## 4 results

**Figure A1:**
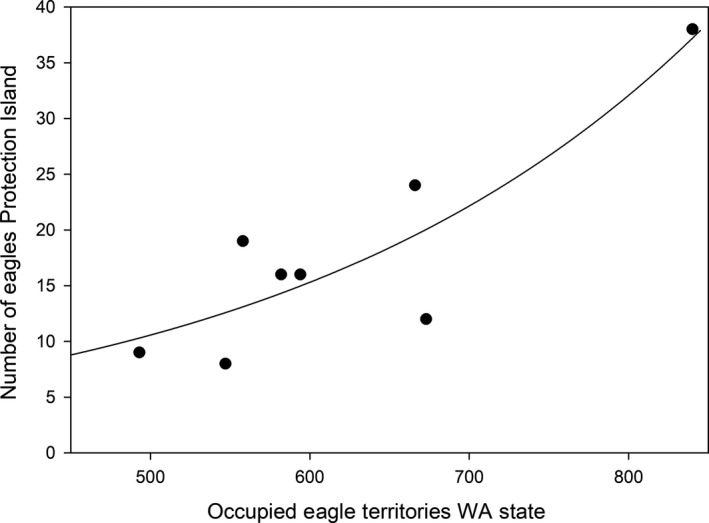
Poisson regression relationship between number of bald eagles on Protection Island and occupied bald eagle territories in Washington State, from Equation (11) in the main text

Numbers of eagles observed on Protection Island were strongly positively correlated with numbers of occupied eagle territories in Washington (*ρ* = 0.86, *p* = 0.006, *n* = 8). Poisson regression produced the relationship

(11) $$\ln\left(\text{PI Eagles}\right)=0.5074+0.003701\left(\text{WA Eagle Territories}\right)$$

(Figure A1) with significant slope coefficient (*p* = 0.0057) but nonsignificant intercept (*p* = 0.42). The estimated dispersion parameter was 1.23, indicating a small amount of overdispersion.

**Figure 3:**
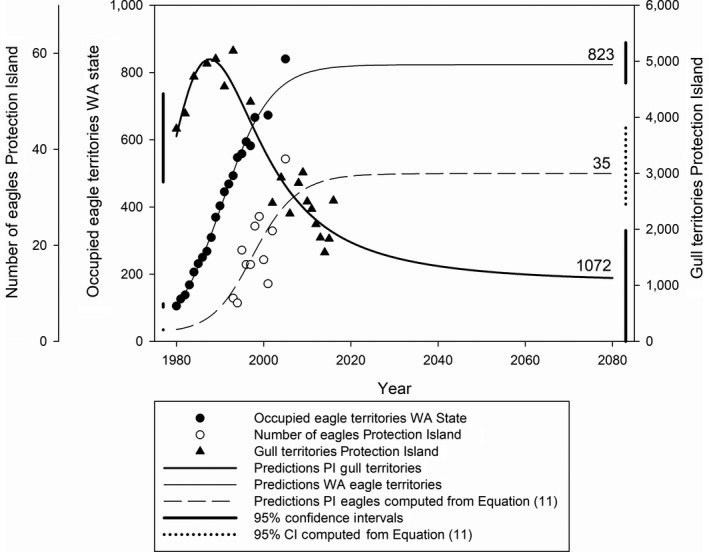
Observed data and model predictions. Observed data (symbols) and predictions of models (2) and (11) from 1980 to 2080 (curves) are numbers of occupied bald eagle territories in Washington State (solid circles, light solid curve), numbers of bald eagles observed at Protection Island, Washington (open circles, dashed curve), and numbers of glaucous‐winged gull nests at the Violet Point colony on Protection Island (triangles, dark solid curve). The 95% confidence intervals for fitted initial conditions and predicted equilibria are marked with vertical lines on the left‐ and right‐hand sides of the graph, respectively

For the fitted point estimates of the parameters (Table 2), the relationship *r* > *αC* holds, indicating that the coexistence equilibrium is stable. Parameter *β* is effectively equal to zero. Thus, the eagle population is predicted to grow logistically without dependence on the gull population. The model predicts that gull nests on Violet Point will equilibrate at 1,072, in contrast to the estimated carrying capacity of *K* = 7,395 nesting pairs (Table 2). Eagle territories in Washington are predicted to equilibrate at 823 territories, which is equal to the predicted carrying capacity *C* (Table 2). The goodness‐of‐fits indicated that the model explains at least 77% of the variability in the data (*R* ^2^ = 0.819 and *R*_A_^2^ = 0.772). At the predicted equilibrium of 823 eagle territories, Equation (11) predicts an equilibrium of 35 eagle visitors on Protection Island. Fitted model predictions for the years 1980–2080 are shown in Figure 3.

**Table 2:** Point estimates for the parameters in model (2) and the initial conditions, with 95% confidence intervals. Estimates for the equilibria, with 95% confidence intervals

	Estimate	95% CI
Parameter		
*G*_0_	3,663	(2,847, 4,422)
*E*_0_	106.7	(101.8, 112.1)
*r*	0.2327	(0.1044, 0.6642)
*K*	7,395	(6,186, 10,444)
*α*	0.0002417	(0.0001460, 0.0005857)
*s*	0.1809	(0.1682, 0.1935)
*C*	823.1	(768.8, 889.0)
*β*	1.876 × 10^−23^	(2.652 × 10^−63^, 2.082 × 10^−13^)
Equilibrium		
*G* (Gull Nests)	1,072	(0, 1981)
*E* (Eagle Terr)	823.1	(768.8, 889.0)
PI Eagles	34.94	(28.58, 44.59)

**Figure A2:**
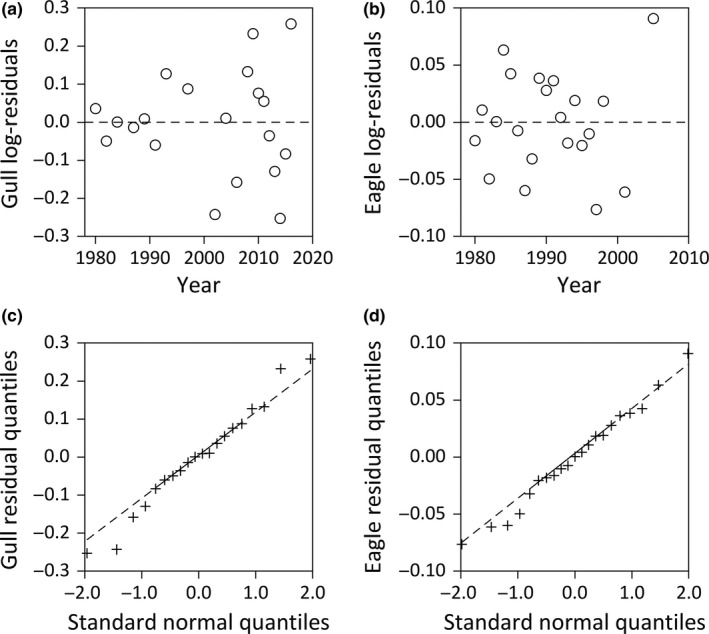
Time series plots of model residuals for (a) glaucous‐winged gulls and (b) bald eagles. Normal quantile‐quantile plots of model residuals for (c) glaucous‐winged gulls and (d) bald eagles

Our analysis of the model residuals for gulls and eagles (Equation (5)) reveals no evidence of significant violations of our model assumptions. The first‐ and second‐order autocorrelation values for gulls were ${\hat{\rho }}_{g}\left(1\right)$ = 0.4580 (*n* = 8, *p* = 0.2538) and ${\hat{\rho }}_{g}\left(2\right)$ = −0.2975 (*n* = 15, *p* = 0.2815). The autocorrelation values for eagles were ${\hat{\rho }}_{e}\left(1\right)$ = 0.1577 (*n* = 18, *p* = 0.5319) and ${\hat{\rho }}_{e}\left(2\right)$ = −0.2771 (*n* = 17, *p* = 0.2817). Neither of the Shapiro–Wilk test statistics for gulls and eagles were significant: *W_g_* = 0.9875 (*p* = 0.9134) for gulls and *W_e_* = 0.9863 (*p* = 0.9862) for eagles. However, the power of these tests is limited due to small sample sizes. Time series and normal quantile–quantile plots of the model residuals appear in Figure A2.

**Figure A3:**
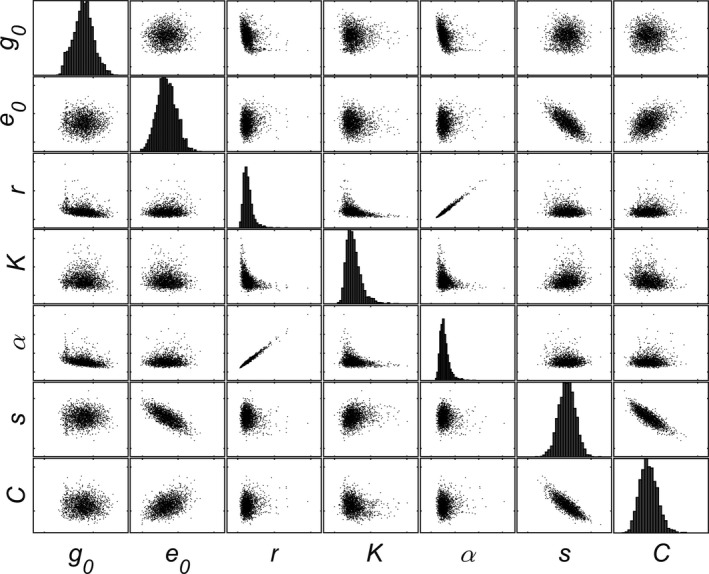
Scatterplot matrix of the 2,000 bootstrapped parameter estimates, excluding *β,* which was always close to zero

A scatterplot matrix of the bootstrapped parameter estimates, excluding *β*, which was always close to zero, is shown in Figure A3. The diagonal plots are histograms showing the distribution of the 2,000 estimates for each parameter. None of these histograms suggest unusual properties for the distributions such as high skewness or multiple modes. The off‐diagonal scatter plots show the pairwise relationships between the parameters for the 2,000 estimated parameter vectors. These plots can reveal strong or unusual dependencies between parameter estimates, as is the case for parameters *r* and* α*. This suggests that large estimates of gull population growth rates coincide with large estimates of gull predation rates, and vice versa.

**Figure 4:**
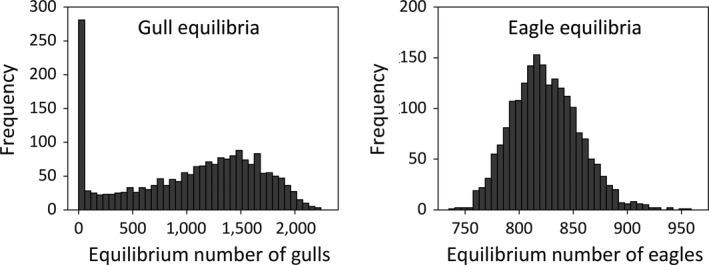
Histograms of the equilibria for both species, based on 2,000 bootstrapped parameter estimates

**Figure 5:**
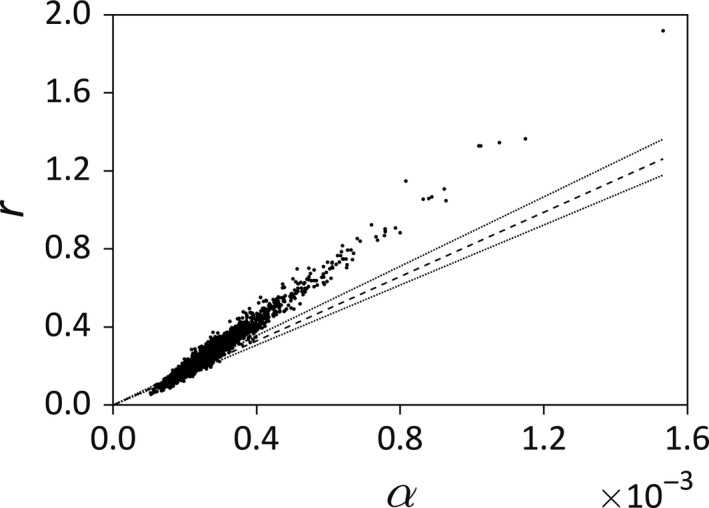
Scatter plot of the 2,000 estimates of *r* versus *α* with the coexistence cutoff condition *r* = *αC* for the point estimate of *C* appearing as a dashed line. Estimates below that line lead to gull colony extinction. The dotted lines are *r* = *αC* using the lower and upper 95% CI bounds for parameter *C*. The area between the dotted lines could be considered an “uncertainty parameter region” for coexistence

In 13.4% of cases, the vector of parameter estimates predicted gull colony extinction. Predicted equilibria were derived for each of the 2,000 bootstrapped parameter vectors, and plotted for both species (Figure 4). The scatter plot of the 2,000 estimates shows *r* versus *α* with the coexistence cutoff condition *r* = *αC* for the point estimate of *C* appearing as a dashed line (Figure 5). Estimates below that line lead to gull colony extinction. The dotted lines are *r* = *αC* using the lower and upper 95% CI bounds for parameter *C*. The area between the dotted lines could be considered an “uncertainty parameter region” for coexistence.

Although our deterministic model cannot predict a time to extinction, we can compute the amount of time it takes gull numbers to fall below a threshold in those cases (267 of 2,000) for which the bootstrapped parameter vectors predict extinction. If we define the threshold as 10% of the estimated carrying capacity for gulls, the estimated mean for the year in which extinction occurs is 2039 with a 95% confidence interval of (2022, 2059). For a threshold of 5% of *K*, the mean is 2073 with a 95% confidence interval of (2040, 2122).

## 5 discussion

We have demonstrated a strong dynamic relationship between the bald eagle population in Washington State and numbers of glaucous‐winged gull nests on Protection Island's Violet Point colony. This relationship exhibits a Lotka–Volterra‐type dynamic that, at the point estimates of the parameters, predicts long‐term coexistence and equilibrium for the two species. The model does not predict predator–prey oscillations as depicted originally by Lotka (1925, 1932) and Volterra (1926). It is notable, however, that the model predicts gull colony extinction for some parameters within the 95% confidence intervals about the point estimates.

The carrying capacity estimate for Washington eagle occupied territories (823) is currently approximately realized and perhaps has been exceeded. There are now more than 1,000 nesting territories in Washington State although the nests are not all active during the same years. The most recent comprehensive survey results show that in 2001 there were 923 territories checked and 705 found to be occupied, and in 2005, the numbers increased to 1,158 territories checked and 893 occupied (J. W. Watson, unpublished data).

Eagles have nested on Protection Island since at least the 1920s (Cowles & Hayward, 2008), and one or two eaglets were raised from a nest located on the island during many years since the early 1980 s (Hayward et al., 2010). Large numbers of transient eagles are attracted to the island each breeding season. For example, on 4 July 2004, 53 eagles were counted during a boat trip around the island (Neil Holcomb, U.S. Fish and Wildlife Service volunteer, personal communication). The number of eagles present on Protection Island typically peaks each year during the second week of July, when gull chicks are hatching and seal afterbirths and dead pups are most abundant (Hayward et al., 2010). Transient adults sometimes are chased back to the mainland by adult residents (unpublished observations).

The impact of bald eagles on seabirds has become increasingly apparent as populations have recovered and some marine fish populations on which they feed have declined (Anderson, Bower, Nysewander, Evenson, & Lovvorn, 2009; Anderson, Lovvorn, Esler, Boyd, & Stick, 2009; Stick & Lindquist, 2009; Therriault, Hay, & Schweigert, 2009). Along the west coast of North America, bald eagles have been implicated as being responsible for declines in local populations of common murres (*Uria aalge*; Parrish et al., 2001; Hipfner, Morrison, & Darvill, 2011), double‐crested cormorants (*Phalacrocorax auritus*, Chatwin, Mather, & Giesbrecht, 2002; Harris, Wilson, & Elliott, 2005), pelagic cormorants (*P. pelagicus*, Chatwin et al., 2002; Harris et al., 2005; Carter, Hebert, & Clarkson, 2009), great blue herons (*Ardea herodias*; Vennesland & Butler, 2004), western grebes (*Aechmophorus occidentalis*; Bower, 2009), and glaucous‐winged gulls (Hayward et al., 2010; Sullivan et al., 2002). The declines have been dramatic in some places. For example, a 131‐km stretch along the coast of Oregon formerly supported more than 380,000 breeding pairs of common murres, but successful reproduction by these birds today is virtually nonexistent. Entire colonies have been abandoned. Murres that remain in colonies harassed by bald eagles typically give up on the breeding process before completing the nesting season (Hipfner et al., 2012). Similar effects on seabirds have been noted in Northern Europe where populations of white‐tailed eagles (*H. albicilla*) have rebounded from declines (Hipfner et al., 2012).

The decline in numbers of glaucous‐winged gulls on Violet Point, Protection Island, paralleled declines and eventual extirpation of double‐crested and pelagic cormorants nesting on Protection Island. Two colonies containing several hundred pairs of double‐crested cormorants thrived on Protection Island for many years, and a small pelagic cormorant colony existed there for several years, but eagle disturbances frequently caused colony residents to flee their nests; by 2007 all three colonies were vacant and have remained so (J. L. Hayward, unpublished data). Rhinoceros auklets (*Cerorhinca monocerata*) remain abundant breeders on Protection Island (Pearson, Hodum, Good, Schrimpf, & Knapp, 2013), although in 2001 they comprised the most common remains beneath an active bald eagle nest on the island (Hayward et al., 2010). Our informal observations suggest that auklets are preyed upon mostly during predawn hours when auklets leave their nest burrows to forage.

Although the model predicts coexistence at the point parameter estimates, the 95% confidence intervals include the possibility of extinction. Of the 2,000 bootstrapped parameter vectors, extinction was predicted in 13.4% of the cases. When we limit our prediction to the 1,788 cases in which the elements of the bootstrapped parameter triplets (*r*,*α,C*) are inside their 95% confidence intervals, then in 11.0% of the cases gull colony extinction was predicted. Thus, for some parameter values within the 95% confidence intervals, our model predicts that the Violet Point gull colony on Protection Island will disappear. If we define the threshold for extinction as 10% of the estimated carrying capacity for gulls, the estimated mean for the year in which extinction occurs is 2039 with a 95% confidence interval of (2022, 2059). For a threshold of 5%, the mean is 2073 with a 95% confidence interval of (2040, 2122). In fact, extinction did occur on Colville Island, located 33 km north of Protection Island. The Colville glaucous‐winged gull colony grew from 1,273 pairs in 1963 to 1,808 pairs in 1975 (Amlaner, Hayward, Schwab, & Stout, 1977; Thoresen & Galusha, 1971). By 2000, however, only ~20 pairs nested on Colville, and more recent observations suggest that nesting gulls are absent from the island (J. L. Hayward, unpublished data). Bald eagles were known to disturb and prey on these gulls during the 1970 s (Hayward et al., 1977), although it is unknown whether this was the cause of colony abandonment.

Extinction of the Protection Island gull colony would impact the local ecosystem in a variety of ways. The effects on vegetation would be pronounced. Gulls physically alter vegetation in their colonies through trampling, digging of nest scrapes, collection of nest material, and disturbance during boundary disputes; they chemically alter the soil through defecation and regurgitation of nondigestible components of food (Ellis, Fariña, & Witman, 2006; Lindborg, Ledbetter, Walat, & Moffett, 2012; Sobey & Kenworthy, 1979); and the decomposition of adult and juvenile gull carcasses on breeding colonies contributes nutrients to the soil (Emslie & Messenger, 1991; Lord & Burger, 1984). Thus, extinction of the gull colony would result in significant changes in the vegetation and in organisms that depend on that vegetation (Sobey & Kenworthy, 1979), and in the loss of a nutrient subsidy to the waters surrounding Protection Island (Hutchinson, 1950; Leentvaar, 1967; McColl & Burger, 1976). Extinction also would eliminate a significant local food source for bald eagles, although gulls are not their only island food (Hayward et al., 2010), and bald eagles may gradually relocate in response to dwindling historic sources of prey (McClelland et al., 1994).

Factors other than the direct and indirect effects of eagles also may impact gull populations. Gulls are scavengers and gull populations increased dramatically during most of the twentieth century (Amlaner et al., 1977; Duhem, Roche, Vidal, & Tatoni, 2008; Kadlec & Drury, 1968; Sullivan et al., 2002). Closure of landfills in this and other regions worldwide has been associated with sharply reduced gull populations (Payo‐Payo et al., 2015). Indeed, the 1992 closure of the Coupeville Landfill (Anonymous, 2001), a popular feeding site for gulls located 19 km northeast of the Violet Point gull colony (Schmidt, 1986), was followed by a 10‐year decline in gull nest counts on Violet Point (Figure 3). Declines in forage fish populations (Blight et al., 2015; McKechnie et al., 2014; Therriault et al., 2009) may have played a role in gull declines. Gulls nest only along the edges of dune grass (*Leymus mollis*), so increases in cover by this plant could impact the size of the colony. Dune grass cover increased from 2.5 ha (14% of Violet Point) in 1980 to 6.6 ha (39% of Violet Point) by 2009 (Cowles et al., 2012). Considerable area suitable for nesting, however, remains unoccupied suggesting that dune grass is not a limiting factor. Increasing sea surface temperatures (SSTs) that occur with El Niño events and climate change have been implicated as a factor that increases gull egg cannibalism and decreases gull colony reproductive output (Hayward et al., 2014). We do not know whether the effects of increasing SSTs interact in some way with eagle effects on the gull population. Although these various confounding factors, which are not included explicitly in our model, undoubtedly contributed to the decline in gull numbers, it is important to note that the gull dynamics nevertheless are well predicted by the model. This suggests that eagle dynamics are one of the most explanatory factors involved in the decline of gulls on Protection Island.

The data used here raised challenges for model parameter estimation. While many sophisticated techniques have been developed to deal with deficiencies in ecological time series data (e.g., Clark & Bjørnstad, 2004; Clark, 2007), our goal was to obtain a reasonable fit of the model to the data and focus on the implications of the model predictions for wildlife management. We scaled population numbers by their standard deviations and used the sum of the root mean squares of the model residuals (Equation(6)) as the objective function for ordinary least squares (OLS) minimization. Some of the estimation issues can be mitigated if one assumes that one of the equations is decoupled from the other, as is the case when *β* = 0. In Appendix B, we present a second method, based on this assumption, where we use OLS parameter estimation on the unscaled population data separately for each species. The resulting parameter estimates (shown in Table A1) are nearly identical to those in Table 2.

Another issue with the parameter estimation and bootstrapping methods is a lack of independence of the model residuals. This occurs because we are directly fitting the model to time series data using OLS. An alternate approach to parameter estimation is to take advantage of transitions between consecutive model states using conditional least squares (CLS). For this method, one uses parameter values and the observed population numbers at a given time to predict the population values in the following time interval (see, e.g., Dennis, Desharnais, Cushing, & Costantino, 1995). One repeats this procedure for all time steps. The best set of parameter estimates are ones that minimize the sum of the squared deviations between the observed numbers and the one‐step predictions. This approach takes advantage of the Markov assumption implicit in the ordinary differential equation model: Future states depend only on the present, not the past. Since the data used for parameter estimation are conditional one‐step transitions, the “observations” are, by assumption, independent. If the population data are not spaced evenly in time, however, then one cannot assume that the random one‐step deviations are identically distributed, since the variance will, in general, depend on the length of the time step. Also, if one does not have observations for all the state space variables at the same times, then one‐step predictions are not possible. Given that both situations exist for the gull and eagle data, we were unable to use the CLS method. More complex methods, such as Bayesian state space modeling and Gibbs sampling, might mitigate these data deficiencies (Clark & Bjørnstad, 2004). Nevertheless, our OLS and bootstrapping procedures provided parameter estimates with a good visual fit to the data and model residuals that showed no evidence of autocorrelation or deviations from normality. The techniques we used are not specific to the predator–prey system we analyzed and could prove useful in other situations where the ecological data provide similar challenges.

**Figure A4:**
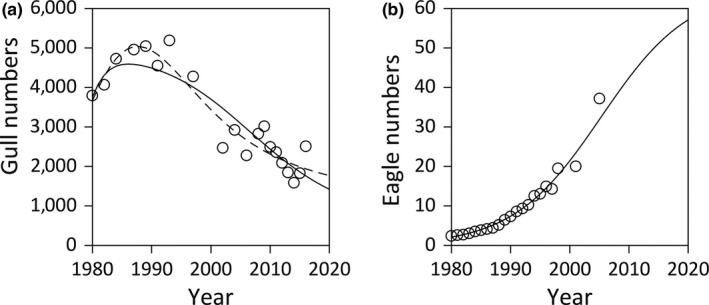
Time series plots of observed (circles) and model‐predicted numbers for (a) glaucous‐winged gulls and (b) bald eagles based on the regression‐predicted eagle numbers for Protection Island. The dashed curve in panel A is the prediction for gulls based on the statewide data

**Figure A5:**
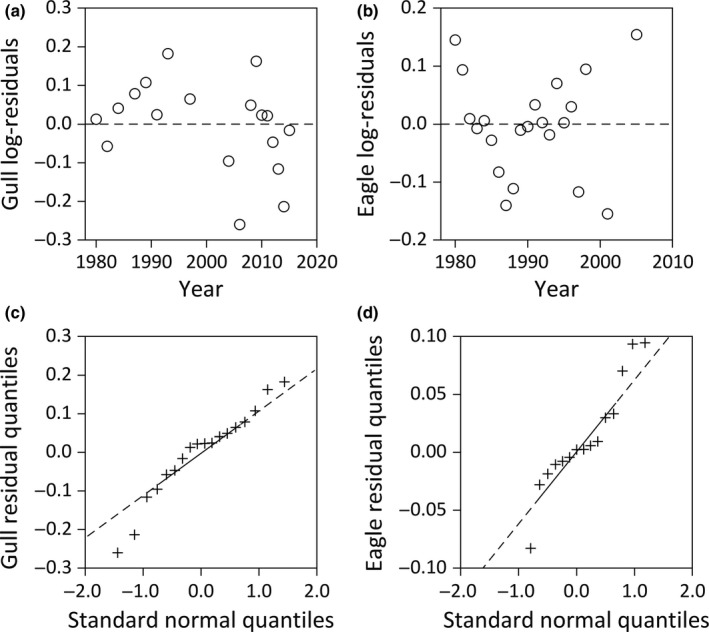
Time series plots of model residuals for (a) glaucous‐winged gulls and (b) bald eagles based on the regression‐predicted eagle numbers for Protection Island. Normal quantile–quantile plots of model residuals for (c) glaucous‐winged gulls and (d) bald eagles. Compare to Figure A2

Our parameter estimation and model predictions for gulls on Protection Island are based on statewide eagle data. This is because these data are more frequent and consistent over time than the eagle observations for Protection Island. However, the Poisson regression can be used to predict eagle numbers on the island based on the statewide data. We repeated our parameter estimation procedure using the predicted island eagle numbers from the nonlinear Equation (4). The estimated parameter values appear in the Table A1. The overall fit was slightly poorer with generalized coefficients of determination of *R* ^2^ = 0.789 and *R*_A_^2^ = 0.735. A graphical comparison of the model fits for the regression‐predicted eagle numbers versus statewide data (Figure A4) suggests that the former underestimates observed gull numbers for the time period 1984–1997. A graphical analysis of the residuals supports this observation and suggests some consistent departures from normality (Figure A5). Moreover, the analysis for the regression‐predicted eagle numbers does not take into account the error associated with the regression predictions, which, based on Figure A1, could be substantial. For these reasons, we feel that the parameter estimation and model analyses based on the statewide eagle data are more reliable.

## 6 CONCLUSIONS

We have shown that the dynamics of a glaucous‐winged gull colony on Protection Island from 1980–2016 can be explained by the number of occupied bald eagle territories in Washington with generalized *R* ^2^ = 0.82. This supports the hypothesis that the rise and decline in gull numbers observed on Protection Island are due largely to the decline and recovery of the bald eagle population. We also have shown that, with 95% confidence, the long‐term dynamic predictions include coexistence but also the possibility that the gull colony will disappear, as occurred on Colville Island.

This study serves as a reminder that the necessary and successful management of one species can have direct and dramatic effects on other species; and it illustrates the uncertainty of those effects. It serves as a cautionary exploration of the future, not only for gulls on Protection Island, but for other seabirds in the Salish Sea. In particular, managers should monitor the numbers of nests in seabird colonies as well as the eagle activity within the colonies to document trends that may lead to colony extinction.

## CONFLICT OF INTEREST

None declared.

## AUTHORS' CONTRIBUTIONS

S. M. H. and E. T. F. posed the mathematical model. R. A. D. and S. M. H. conducted statistical model fitting and time series analysis. J. W. W. provided eagle data. J. G. G. and J. L. H. provided gull data. All authors contributed to the writing of the manuscript. All authors gave final approval for publication.

## Data Availability

The data are available in Table 1 of this manuscript.

## APPENDIX A Model Analysis

### EQUILIBRIA

The equilibria of model (2) are found by setting the derivative to zero in each differential equation, factoring the resulting system of algebraic equations to obtain

$$\begin{array}{c} G\left(r-\frac{r}{K}G-\alpha E\right)=0\\ E\left(s-\frac{s}{C}E+\beta G\right)=0 \end{array},$$

and solving for the state variables *G* and *E*. This produces ordered pairs (*G*,* E*) that satisfy both equations simultaneously. One can check that there are exactly four possible equilibrium pairs: (0, 0), (K, 0), (0, C), and ($\overset{¯}{G},\overset{¯}{E}$), with

$$\overset{¯}{G}=\frac{sK\left(r-\alpha C\right)}{\alpha \beta KC+rs}\;\text{and}\;\overset{¯}{E}=\frac{rC\left(s+\beta K\right)}{\alpha \beta KC+rs}.$$

### BIOLOGICALLY FEASIBLE EQUILIBRIA

An equilibrium (*G*,* E*) is biologically feasible if both *G* ≥ 0 and *E* ≥ 0. The equilibria (0, 0), (*K*, 0), and (0, *C*) are therefore always biologically feasible. It is straightforward to check that the coexistence equilibrium ($\overset{¯}{G},\overset{¯}{E}$) is biologically feasible if and only if *r* ≥ *αC*. If equality holds, that is if *r* = *αC*, then ($\overset{¯}{G},\overset{¯}{E}$) collapses to the (0,*C*) equilibrium. We say that ($\overset{¯}{G},\overset{¯}{E}$) is *positive* if and only if

(A1) r>αC.

Thus, the coexistence equilibrium is positive if and only if the inherent growth rate *r* of the gull population is greater than the rate at which gulls can be taken by *C* eagle pairs.

### STABILITY OF THE EQUILIBRIA

The stability of each equilibrium pair is determined by the process of linearization. A comprehensive overview of linearization and stability analysis is found in Henson (2012). Linearization involves computing the Jacobian matrix

$$J\left(G,E\right)=\left[\begin{array}{cc} \begin{array}{c} r-\frac{2r}{K}G-\alpha E \end{array} & -\alpha G\\ \beta E & s-\frac{2s}{C}E+\beta G \end{array}\right]$$

at the equilibrium pair and finding its eigenvalues. If both eigenvalues of Jacobian matrix are negative real numbers, then the equilibrium is called a stable node and nearby solutions approach it in a nonoscillatory fashion. If the eigenvalues are a complex conjugate pair with negative real part, then the equilibrium is called a stable spiral, and nearby solutions approach it in an oscillatory fashion. If at least one of the eigenvalues is positive or if the eigenvalues are a complex conjugate pair with positive real part, then the equilibrium is unstable.

At the equilibrium (0, 0), the Jacobian matrix has two positive eigenvalues *λ* = *r* and *λ* = *s*; hence, the equilibrium solution (0, 0) is always unstable. At equilibrium (*K*, 0), the Jacobian has one positive eigenvalue, *λ* = s + *βK*, and one negative eigenvalue, *λ* = −*r*. Therefore, the equilibrium solution (*K*, 0) is also unstable.

At equilibrium (0, *C*), the Jacobian has one eigenvalue that is always negative, *λ* = −*s*, and one eigenvalue that can be either positive or negative, *λ = r − *αC. If condition (A1) holds, the second eigenvalue is positive, so the equilibrium (0, *C*) is unstable. Note that the coexistence equilibrium solution ($\overset{¯}{G},\overset{¯}{E}$) is positive only if (0, *C*) is unstable.

At the coexistence equilibrium ($\overset{¯}{G},\overset{¯}{E}$), the eigenvalues are

$$\lambda =\frac{-\left[\left(r-\alpha C)+(s+\beta K\right)\right]\pm \sqrt{{\left[\left(r-\alpha C)+(s+\beta K\right)\right]}^{2}-4\left(r-\alpha C\right)\left(s+\beta K\right)\left(1+\frac{\alpha \beta KC}{\mathrm{rs}}\right)}}{2\;\left(1+\frac{\alpha \beta KC}{\mathrm{rs}}\right)}.$$

If (A1) holds, then both eigenvalues are negative (if real) or have negative real part (if complex). Thus, if the coexistence equilibrium ($\overset{¯}{G},\overset{¯}{E}$) is positive, then it is stable. Whether it is a stable node or stable spiral depends on the parameter values.

### SUMMARY

If *r* > *αC*, then all four equilibrium pairs are biologically feasible. The equilibria (0, 0), (*K*, 0), and (0, C) are unstable, and the coexistence equilibrium ($\overset{¯}{G},\overset{¯}{E}$) is positive and stable. The system will approach coexistence either with or without damped oscillations, depending on whether the eigenvalues are complex or real.

If *r* < *αC*, the coexistence equilibrium is not biologically feasible. The equilibria (0, 0) and (K, 0) are unstable and the equilibrium (0, C) is stable. In this case, the gull population will go extinct and the eagle population will approach its carrying capacity.

## APPENDIX B Graphical Analyses of Model Residuals

We conducted a graphical analysis of the model residuals defined in Equation (5). These residuals are the log‐scale deviations from the predicted gull and eagle numbers, obtained using the model (4) with the point estimates of the parameters and the observed values after dividing by their respective standard deviations. The first two panels of Figure A2 show the residuals plotted as a function of time. There is no evidence of systematic departures from zero, although the residuals for gulls are smaller in the earlier years. The last two panels of Figure A2 show normal quantile–quantile plots of the gull and eagle residuals. The dashed line is the normal distribution expectation, and the solid line is the reference line connecting the first and third quartiles. There is no evidence in these plots of large systematic deviations from normality.

## APPENDIX C Parameter Estimation for Decoupled Equations

For the full model (2) in the main text, the parameter *β* is the conversion rate of gulls into eagle births. Our estimate of *β* = 1.876 × 10^−23^ is effectively zero. This might be expected since we are using statewide data for eagles, while gull population numbers are local to Protection Island. If we assume a priori that *β* =0, then we can decouple the equations for eagles from the equation for gulls and estimate the parameters for the eagle population separately. The predicted densities for the eagle population can then be used to find best fitting parameter estimates for gulls. Two advantages of this approach are (a) the population densities do not need to be scaled by the standard deviations to arrive at commensurate numbers for eagles and gulls, and (b) there is one less parameter to be estimated.

We used the decoupled equation approach to obtain a set of parameter estimates for comparison to the estimates in Table 2. Eagle densities can be computed directly using the closed form solution of the logistic equation:

(A2) $$e_{t}=\frac{C}{1+\left(\frac{C-e_{0}}{e_{0}}\right)\exp\left(-st\right)}.$$

We computed residuals between the predicted and observed eagle numbers on a log scale:

(A3) $$\varepsilon_{t}=\ln\left(E_{t}\right)-\ln\left(e_{t}\left(e_{0},s,C\right)\right).$$

We obtained parameter estimates for *e*_0_, *s*, and *C* by minimizing the sum of the squared values for these residuals. We then substituted equation (A2) into the differential equation for the gull population and used numerical integration to obtain population predictions, *g_t_*, for gulls. We computed residuals on the log scale,

(A4) $$\gamma_{t}=\ln\left(G_{t}\right)-\ln\left(g_{t}\left(g_{0},r,K,\alpha \right)\right),$$

and estimated the parameters *g*_0_, *r*,* K,* and *α* by minimizing $\sum \gamma_{t}^{2}$.

Table A1 lists the parameter estimates and coefficients of determination for the statistical method from the main text and the one presented here. For the approach involving the decoupled equations, we scaled the observed and predicted values using the observed standard deviations for the gull and eagle data and computed RMS as given in Equation (6). We used this to compute *R* ^2^ and *R_A_* ^2^ as described in the main text. For Equation (9), the number of estimated parameters was *p* = 8 for the coupled least squares approach and *p* = 7 for the method presented here.

**Table A1:** Point parameter estimates and coefficients of determination for the three methods of parameter estimation (PI = Protection Island)

Parameter or Quantity	Coupled least squares	Decoupled least squares	Using regression‐predicted eagle numbers for PI
*g*_0_	3,663	3,663	3,748
*e*_0_	106.7	106.9	2.119
*r*	0.2327	0.2338	0.5031
*K*	7,395	7,376	4,913
*α*	0.0002417	0.0002419	0.007187
*s*	0.1809	0.1803	0.1333
*C*	823.1	825.4	65.00
*β*	1.876 x 10^‐23^	—	1.580 x 10^‐7^
$\overset{¯}{G}$	1,072	1,076	349.8
$\overset{¯}{E}$	823.1	825.4	65.02
*R* ^2^	81.9%	81.9%	78.9%
*R_A_* ^2^	77.2%	77.9%	73.5%

The parameter estimates for the two methods are nearly identical. They had equivalent goodness‐of‐fits based on the coefficient of determination, *R* ^2^. However, since the decoupled model had one less parameter, it has a slightly higher adjusted coefficient of determination, *R_A_* ^2^.

## References

[ece35011-bib-0001] Amlaner, C. J. , Hayward, J. L. , Schwab, E. R. , & Stout, J. F. (1977). Increases in a population of nesting glaucous‐winged gulls disturbed by humans. The Murrelet, 58, 18–20. 10.2307/3535708

[ece35011-bib-0002] Anderson, E. M. , Bower, J. L. , Nysewander, D. R. , Evenson, J. R. , & Lovvorn, J. R. (2009). Changes in avifaunal abundance in a heavily used wintering and migration site in Puget Sound, Washington, during 1966–2007. Marine Ornithology, 37, 19–27.

[ece35011-bib-0003] Anderson, E. M. , Lovvorn, J. R. , Esler, D. , Boyd, W. S. , & Stick, K. C. (2009). Using predator distributions, diet, and condition to evaluate seasonal foraging sites: Sea ducks and herring spawn. Marine Ecology Progress Series, 386, 287–302. 10.3354/meps08048

[ece35011-bib-0004] Anderson‐Sprecher, R. (1994). Model comparisons and R^2^ . The American Statistician, 48, 113–117.

[ece35011-bib-0005] Anonymous . (2001). Trash talk: waste division heads it up, moves it out. Whidbey News‐Times, 6 June. Accessed online on 2 July 2018, http://www.whidbeynewstimes.com/news/trash-talk-waste-division-heads-it-up-moves-it-out/.

[ece35011-bib-0006] Atkins, N. , & Heneman, B. (1987). The dangers of gill netting to seabirds. American Birds, 41, 1395–1403.

[ece35011-bib-0007] Bell, D. A. (1996). Genetic differentiation, geographic variation and hybridization in gulls of the *Larus glaucescens‐occidentalis* complex. Condor, 98, 527–546. 10.2307/1369566

[ece35011-bib-0008] Bell, D. A. (1997). Hybridization and reproductive performance in gulls of the *Larus glaucescens‐occidentalis* complex. Condor, 99, 585–594. 10.2307/1370471

[ece35011-bib-0009] Blight, L. K. , Drever, M. C. , & Arcese, P. (2015). A century of change in glaucous‐winged gull (*Larus glaucescens*) populations in a dynamic coastal environment. Condor, 117, 108–120.

[ece35011-bib-0010] Bolker, B. M. , Gardner, B. , Maunder, M. , Berg, C. W. , Brooks, M. , Comita, L. , … Zipkin, E. (2013). Strategies for fitting nonlinear ecological models in R, AD Model Builder, and BUGS. Methods in Ecology and Evolution, 4, 501–512. 10.1111/2041-210X.12044

[ece35011-bib-0011] Bower, J. L. (2009). Changes in marine bird abundance in the Salish Sea: 1975 to 2007. Marine Ornithology, 37, 9–17.

[ece35011-bib-0012] Buehler, D. A. (2000). Bald eagle (*Haliaeetus leucocephalus*) In Poole A. F., & Gill F. B. (Eds.), The Birds of North America, No. 506. A. P. a. F. Gill. Philadelphia, PA: The Birds of North America Inc.

[ece35011-bib-0013] Carpenter, S. R. , Cottingham, K. L. , & Stow, C. A. (1994). Fitting predator‐prey models to time series with observation errors. Ecology, 75, 1254–1264.

[ece35011-bib-0014] Carter, H. R. , Hebert, P. N. , & Clarkson, P. V. (2009). Decline of pelagic cormorants in Barkley Sound. British Columbia. Wildlife Afield, 4, 3–32.

[ece35011-bib-0015] Chatwin, T. A. , Mather, M. H. , & Giesbrecht, T. D. (2002). Changes in pelagic and double‐crested cormorant nesting populations in the Strait of Georgia. British Columbia. Northwestern Naturalist, 83, 109–117. 10.2307/3536609

[ece35011-bib-0016] Clark, J. S. (2007). Models for ecological data: An introduction. Princeton, NJ: Princeton University Press.

[ece35011-bib-0017] Clark, J. S. , & Bjørnstad, O. N. (2004). Population time series: Process variability, observation errors, missing values, lags, and hidden states. Ecology, 85, 3140–3150. 10.1890/03-0520

[ece35011-bib-0018] Cowles, D. L. , Galusha, J. G. , & Hayward, J. L. (2012). Negative interspecies interactions in a glaucous‐winged gull colony on Protection Island, Washington. Northwestern Naturalist, 93, 89–100. 10.1898/nwn11-12.1

[ece35011-bib-0019] Cowles, D. L. , & Hayward, J. L. (2008). Historical changes in the physical and vegetational characteristics of Protection Island, Washington. Northwest Science, 82, 174–184. 10.3955/0029-344X-82.3.174

[ece35011-bib-0020] Cushing, J. M. , Costantino, R. F. , Dennis, B. , Desharnais, R. A. , & Henson, S. M. (2003). Chaos in ecology: Experimental nonlinear dynamics. San Diego, CA: Academic Press.

[ece35011-bib-0021] de Valpine, P. , & Hastings, A. (2002). Fitting population models incorporating process noise and observation error. Ecological Monographs, 72, 57–76. 10.1890/0012-9615(2002)072[0057:FPMIPN]2.0.CO;2

[ece35011-bib-0022] DeGange, A. R. , & Nelson, J. W. (1982). Bald eagle predation on nocturnal seabirds. Journal of Field Ornithology, 53, 407–409.

[ece35011-bib-0023] Dennis, B. , Desharnais, R. A. , Cushing, J. M. , & Costantino, R. F. (1995). Nonlinear demographic dynamics: Mathematical models, statistical methods, and biological experiments. Ecological Monographs, 65, 261–282. 10.2307/2937060

[ece35011-bib-0024] Dennis, B. , Desharnais, R. A. , Cushing, J. M. , Henson, S. M. , & Costantino, R. F. (2001). Estimating chaos and complex dynamics in an insect population. Ecological Monographs, 71, 277–303. 10.1890/0012-9615(2001)071[0277:ECACDI]2.0.CO;2

[ece35011-bib-0025] Dennis, B. , Munholland, P. L. , & Scott, J. M. (1991). Estimation of growth and extinction parameters for endangered species. Ecological Monographs, 61, 115–143. 10.2307/1943004

[ece35011-bib-0026] Dennis, B. , Ponciano, J. M. , Lele, S. R. , Taper, M. L. , & Staples, D. F. (2006). Estimating density dependence, process noise, and observation error. Ecological Monographs, 76, 323–341. 10.1890/0012-9615(2006)76[323:EDDPNA]2.0.CO;2

[ece35011-bib-0027] Duhem, C. , Roche, P. K. , Vidal, E. , & Tatoni, T. (2008). Effects of anthropogenic food resources on yellow‐legged gull colony size on Mediterranean islands. Population Ecology, 50, 91–100. 10.1007/s10144-007-0059-z

[ece35011-bib-0028] Elliott, K. H. , Elliott, J. E. , Wilson, L. K. , Jones, I. , & Stenerson, K. (2011). Density‐dependence in the survival and reproduction of bald eagles: Linkages to chum salmon. The Journal of Wildlife Management, 75, 1688–1699. 10.1002/jwmg.233

[ece35011-bib-0029] Ellis, J. C. , Fariña, J. M. , & Witman, J. D. (2006). Nutrient transfer from sea to land: The case of gulls and cormorants in the Gulf of Maine. Journal of Animal Ecology, 75, 565–574. 10.1111/j.1365-2656.2006.01077.x 16638009

[ece35011-bib-0030] Elton, C. , & Nicholson, M. (1942). The ten‐year cycle in numbers of the lynx in Canada. Journal of Animal Ecology, 11, 215–244. 10.2307/1358

[ece35011-bib-0031] Emslie, S. D. , & Messenger, S. L. (1991). Pellet and bone accumulation at a colony of western gulls (*Larus occidentalis*). Journal of Vertebrate Paleontology, 11, 133–136.

[ece35011-bib-0032] Falck, W. , Bjornstad, O. N. , & Stenseth, N. C. (1995). Bootstrap estimated uncertainty of the dominant Lyapunov exponent for Holarctic microtine rodents. Proceedings of the Royal Society of London B, 261, 159–165. 10.1098/rspb.1995.0131 7568270

[ece35011-bib-0033] Galusha, J. G. , & Hayward, J. L. (2002). Bald eagle activity at a gull colony and seal rookery on Protection Island, Washington. Northwestern Naturalist, 83, 23–25. 10.2307/3536511

[ece35011-bib-0034] Galusha, J. G. , Vorvick, B. , Opp, M. R. , & Vorvick, P. T. (1987). Nesting season censuses of seabirds on Protection Island, Washington. The Murrelet, 68, 103–107. 10.2307/3534115

[ece35011-bib-0035] Harris, M. L. , Wilson, L. K. , & Elliott, J. E. . (2005). An assessment of PCBs and OC pesticides in eggs of double‐crested (*Phalacrocorax auritus*) and pelagic (*P. pelagicus*) cormorants from the west coast of Canada, 1970 to 2002. Ecotoxicology, 14, 607–625. 16215696 10.1007/s10646-005-0011-y

[ece35011-bib-0036] Harvey, C. J. , Good, T. P. , & Pearson, S. F. (2012). Top–down influence of resident and overwintering bald eagles (*Haliaeetus leucocephalus*) in a model marine ecosystem. Canadian Journal of Zoology 90:903–914.

[ece35011-bib-0037] Hayward, J. L. (2009). Bald eagle predation on harbor seal pups. Northwestern Naturalist, 90, 51–53. 10.1898/1051-1733-90.1.51

[ece35011-bib-0038] Hayward, J. L. , Galusha, J. G. , & Henson, S. M. (2010). Foraging‐related activity of bald eagles at a Washington seabird colony and seal rookery. Journal of Raptor Research, 44, 19–29. 10.3356/JRR-08-107.1

[ece35011-bib-0039] Hayward, J. L. , Gillett, W. H. , Amlaner, C. J. , & Stout, J. F. (1977). Predation on gulls by bald eagles in Washington. The Auk, 94, 375.

[ece35011-bib-0040] Hayward, J. L. , Weldon, L. M. , Henson, S. M. , Megna, L. C. , Payne, B. G. , & Moncrieff, A. E. (2014). Egg cannibalism in a gull colony increases with sea surface temperature. Condor, 116, 62–73. 10.1650/CONDOR-13-016-R1.1

[ece35011-bib-0041] Henson, S. M. (2012). Phase plane analysis In Hastings A., & Gross L. (Eds.), Encyclopedia of theoretical ecology (p. 538–545). Berkeley, CA: University of California Press.

[ece35011-bib-0042] Henson, S. M. , Weldon, L. M. , Hayward, J. L. , Greene, D. J. , Megna, L. C. , & Serem, M. C. (2012). Coping behaviour as an adaptation to stress: Post‐disturbance preening in colonial seabirds. Journal of Biological Dynamics, 6, 17–37. 10.1080/17513758.2011.605913 22873521

[ece35011-bib-0043] Hipfner, J. M. , Blight, L. K. , Lowe, R. W. , Wilhelm, S. I. , Robertson, G. J. , Barrett, R. T. , … Good, T. P. (2012). Unintended consequences: How the recovery of sea eagle *Haliaeetus* spp. populations in the northern hemisphere is affecting seabirds. Marine Ornithology, 40, 39–52.

[ece35011-bib-0044] Hipfner, J. M. , Morrison, K. W. , & Darvill, R. (2011). Peregrine Falcons enable two species of colonial seabirds to breed successfully by excluding other aerial predators. Waterbirds, 34, 82–88. 10.1675/063.034.0110

[ece35011-bib-0045] Hutchinson, G. E. (1950). Survey of contemporary knowledge of biogeochemistry, 3, The biogeochemistry of vertebrate excretion. Bulletin of the American Museum of Natural History, 96, 34.

[ece35011-bib-0046] Kadlec, J. , & Drury, W. H. (1968). Structure of the New England herring gull population. Ecology, 49, 645–676. 10.2307/1935530

[ece35011-bib-0047] Leentvaar, P. (1967). Observations on guanotrophic environments. Hydrobiologia, 29, 441–489.

[ece35011-bib-0048] Lindborg, V. A. , Ledbetter, J. F. , Walat, J. M. , & Moffett, C. (2012). Plastic consumption and diet of glaucous‐winged gulls (*Larus glaucescens*). Marine Pollution Bulletin, 64, 2351–2356. 10.1016/j.marpolbul.2012.08.020 22995785

[ece35011-bib-0049] Lord, W. D. , & Burger, J. F. (1984). Arthropods associated with herring gull (*Larus argentatus*) and great black‐backed gull (*Larus marinus*) carrion on islands in the Gulf of Maine. Environmental Entomology, 13, 1261–1268.

[ece35011-bib-0050] Lotka, A. J. (1925). Elements of physical biology. Baltimore, MD: Williams and Wilkins.

[ece35011-bib-0051] Lotka, A. J. (1932). The growth of mixed populations: Two species competing for a common food supply. Journal of the Washington Academy of Science, 22, 461–469.

[ece35011-bib-0052] McClelland, B. R. , Young, L. S. , McClelland, P. T. , Crenshaw, J. G. , Allen, H. L. , & Shea, D. S. (1994). Migration ecology of bald eagles from autumn concentrations in Glacier National Park, Montana. Wildlife Monograph, 125, 3850–61.

[ece35011-bib-0053] McColl, J. G. , & Burger, J. (1976). Chemical inputs by a colony of Franklin's gulls nesting in cattails. American Midland Naturalist, 96, 270–280. 10.2307/2424068

[ece35011-bib-0054] McKechnie, I. , Lepofsky, D. , Moss, M. L. , Butler, V. L. , Orchard, T. J. , Coupland, G. , … Lertzman, K. (2014). Archaeological data provide alternative hypotheses on Pacific herring (*Clupea pallasii*) distribution, abundance, and variability. Proceedings of the National Academy of Sciences of the United States of America, 111, E807–E816. 24550468 10.1073/pnas.1316072111 PMC3948274

[ece35011-bib-0055] Megna, L. C. , Moncrieff, A. E. , Hayward, J. L. , & Henson, S. M. (2014). Equal reproductive success of phenotypes in the *Larus glaucescens‐occidentalis* complex. Journal of Avian Biology, 45, 410–416.

[ece35011-bib-0056] Middleton, H. A. , Butler, R. W. , & Davidson, P. (2018). Waterbirds alter their distribution and behavior in the presence of bald eagles (*Haliaeetus leucocephalus*). Northwestern Naturalist, 99, 21–30.

[ece35011-bib-0057] Millar, J. G. , & Lynch, D. (2006). U.S.D.I. Endangered and Threatened wildlife and plants: Removing the bald eagle in the lower 48 states from the list of endangered and threatened wildlife. U.S.D.I. Fish and Wildlife Service. Federal Register, 71(32), 8238–8251.

[ece35011-bib-0058] Moncrieff, A. E. , Megna, L. C. , Hayward, J. L. , & Henson, S. M. (2013). Mating patterns and breeding success in gulls of the *Larus glaucescens‐occidentalist* complex, Protection Island, Washington, USA. Northwestern Naturalist, 94, 67–75.

[ece35011-bib-0059] Moul, I. E. , & Gebauer, M. B. (2002). Status of the double‐crested cormorant in British Columbia, B. C. Minist. Water, Land and Air Protection, Biodiversity Branch, Victoria, BC. Wildl. Working Rep. No. WR‐105, 36 p.

[ece35011-bib-0060] Parrish, J. K. , Marvier, M. , & Paine, R. T. (2001). Direct and indirect effects: Interactions between bald eagles and common murres. Ecological Applications, 11, 1858–1869. 10.1890/1051-0761(2001)011[1858:DAIEIB]2.0.CO;2

[ece35011-bib-0061] Payo‐Payo, A. , Oro, D. , Igual, J. M. , Jover, L. , Sanpera, C. , & Taveccia, G. (2015). Population control of an overabundant species achieved through consecutive anthropogenic perturbations. Ecological Applications, 25, 2228–2239. 10.1890/14-2090.1 26910951

[ece35011-bib-0062] Pearson, S. F. , Hodum, P. J. , Good, T. P. , Schrimpf, M. , & Knapp, S. M. (2013). A model approach for estimating colony size, trends, and babitat associations of burrow‐nesting seabirds. Condor, 115, 356–365.

[ece35011-bib-0063] Power, E. A. (1976). Protection Island and the Power family. Unpublished manuscript, Jefferson County, WA: Historical Society Archives.

[ece35011-bib-0064] Richardson, F. (1961). Breeding biology of the rhinoceros auklet on Protection Island, Washington. Condor, 63, 456–473. 10.2307/1365278

[ece35011-bib-0065] Ricklefs, R. E. (1990). Ecology, 3rd ed. (p. 497). New York, NY: W. H. Freeman and Company.

[ece35011-bib-0066] Schmidt, D. F. (1986). The numbers, distribution and behavior of glaucous‐winged gulls (Larus glaucescens) at a sanitary landfill. M.S. thesis. Walla Walla College, 42 p.

[ece35011-bib-0067] Shapiro, S. S. , & Wilk, M. B. (1965). An analysis of variance test for normality (complete samples). Biometrika, 52, 591–611. 10.1093/biomet/52.3-4.591

[ece35011-bib-0068] Sobey, D. G. , & Kenworthy, J. B. (1979). The relationship between herring gulls and the vegetation of their breeding colonies. Journal of Ecology, 67, 469–496. 10.2307/2259108

[ece35011-bib-0069] Stalmaster, M. V. (1987). The bald eagle. New York, NY: Universe Books.

[ece35011-bib-0070] Stick, K. C. , & Lindquist, A. (2009). 2008 Washington State Herring Stock Status Report. Olympia, WA: Washington Department of Fish and Wildlife, Fish Program, Fish Management Division.

[ece35011-bib-0071] Stinson, D. W. , Watson, J. W. , & McAllister, K. R. (2001). Washington state status report for the bald eagle (p. 92). Fish and Wildlife, Olympia: Washington Department.

[ece35011-bib-0072] Sullivan, T. M. , Hazlitt, S. L. , & Lemon, M. J. F. (2002). Population trends of nesting glaucous‐winged gulls, *Larus glaucescens*, in the southern Strait of Georgia, British Columbia. The Canadian Field‐Naturalist, 116, 603–606.

[ece35011-bib-0073] Therriault, T. W. , Hay, D. E. , & Schweigert, J. F. (2009). Biological overview and trends in pelagic forage fish abundance in the Salish Sea (Strait of Georgia, British Columbia). Marine Ornithology, 37, 3–8.

[ece35011-bib-0074] Thoresen, A. C. , & Galusha, J. G. (1971). A nesting population study of some islands in the Puget Sound area. The Murrelet, 52, 20–23. 10.2307/3534522

[ece35011-bib-0075] Vennesland, R. G. , & Butler, R. W. (2004). Factors influencing great blue heron nesting productivity on the Pacific Coast of Canada from 1998 to 1999. Waterbirds, 27, 289–296. 10.1675/1524-4695(2004)027[0289:FIGBHN]2.0.CO;2

[ece35011-bib-0076] Volterra, V. (1926). Variazioni e fluttuazioni del numero d'individui in specie animali conviventi. Memoria Della Reale Accademia Nazionale Dei Lincei, 2, 31–113.

[ece35011-bib-0077] Watson, J. W. (2002). Comparative home ranges and food habits of bald eagles nesting in four aquatic habitats in western Washington. Northwestern Naturalist, 83, 101–108. 10.2307/3536608

[ece35011-bib-0078] Watson, J. W. , Stinson, D. W. , McAllister, K. R. , & Owens, T. E. (2002). Population status of bald eagles breeding in Washington at the end of the 20th century. Journal of Raptor Research, 36, 161–169.

[ece35011-bib-0079] White, A. F. , Heath, J. P. , & Gisborne, B. (2006). Seasonal timing of bald eagle attendance and influence on activity budgets of glaucous‐winged gulls in Barkley Sound, British Columbia. Waterbirds, 29, 497–500. 10.1675/1524-4695(2006)29[497:STOBEA]2.0.CO;2

[ece35011-bib-0080] Zar, J. H. (2009). Biostatistical analysis, 5th ed Upper Saddle River, NJ: Prentice Hall.

